# A Novel Approach Based on Bipartite Network Recommendation and KATZ Model to Predict Potential Micro-Disease Associations

**DOI:** 10.3389/fgene.2019.01147

**Published:** 2019-11-15

**Authors:** Shiru Li, Minzhu Xie, Xinqiu Liu

**Affiliations:** ^1^College of Information Science and Engineering, Hunan Normal University, Changsha, China; ^2^Hunan Vocational College of Engineering, Changsha, China

**Keywords:** microbe, disease, KATZ model, bipartite network recommendation, Gaussian interaction profile kernel similarity

## Abstract

Accumulating evidence indicates that the microbes colonizing human bodies have crucial effects on human health and the discovery of disease-related microbes will promote the discovery of biomarkers and drugs for the prevention, diagnosis, treatment, and prognosis of diseases. However clinical experiments of disease-microbe associations are time-consuming, laborious and expensive, and there are few methods for predicting potential microbe-disease association. Therefore, developing effective computational models utilizing the accumulated public data of clinically validated microbe-disease associations to identify novel disease-microbe associations is of practical importance. We propose a novel method based on the KATZ model and Bipartite Network Recommendation Algorithm (KATZBNRA) to discover potential associations between microbes and diseases. We calculate the Gaussian interaction profile kernel similarity of diseases and microbes based on validated disease-microbe associations. Then, we construct a bipartite graph and execute a bipartite network recommendation algorithm. Finally, we integrate the disease similarity, microbe similarity and bipartite network recommendation score to obtain the final score, which is used to infer whether there are some novel disease-microbe interactions. To evaluate the predictive power of KATZBNRA, we tested it with the walk length 2 using global leave-one-out cross validation (LOOV), two-fold and five-fold cross validations, with AUCs of 0.9098, 0.8463 and 0.8969, respectively. The test results also show that KATZBNRA is more accurate than two recent similar methods KATZHMDA and BNPMDA.

## Introduction

A microbe is a microscopic organism, including bacteria, eukaryotes, archaea, and viruses (Wu et al., 2018). Various types of microbes live on or in different parts of a human body such as the skin, mouth, hair, stomach, and gastrointestinal tract. An adult human body contains a large number of bacterial cells, which is estimated to reach 10^14^ and much more than the total number of human cells, with more than 5 million microbe genes, outnumbering the human genes by more than 100 fold (Sommer and Backhed, 2013). Most microbes are harmless and some are beneficial to humans (Grice and Segre, 2011). Recently, accumulated experimental evidence shows that microbes have an important impact on human health, nutrient absorption, immune response, cancer control, and the prevention of pathogen colonization (Wu et al., 2018). For example, the gut microbiota could significantly contribute nutrition absorption by producing indispensable vitamins and decomposing indigestible polysaccharides, and it also has an important impact on the mucus layer, the balance of antimicrobial peptides, and immunoglobulin A, and the differentiation and activation of some lymphocyte populations (Sommer and Backhed, 2013). Therefore, the gut microbiota is thought to be an extra ‘organ’ of humans (Gill et al., 2006). But some microbes may contribute to disease, such as psoriasis and inflammatory bowel disease (IBD). There have been reports that psoriasis occurs after strep throat and could worsen due to the colonization of Candida albicans, Malassezia, and Staphylococcus aureus on the skin or in the gut (Fry and Baker, 2007). Aroniadis et. al. (Aroniadis and Brandt, 2013) indicated that the biodiversity of bacteria, such as Bacteroidetes and Firmicutes, colonizing in individuals affected by IBD has been found to be reduced by 30 to 50%. Wang et. al. (Wang and Jia, 2016) showed that the gut microbiota’s dysbiosis might be a key environmental risk factor of many human diseases, though it’s difficult to reveal the true causality.

To explore the relationship between microbes and their human hosts, scientists from many countries collaborated and launched the Human Microbiome Project (HMP) (Human Microbiome Project, 2012a). Recently, high-throughput sequencing techniques and corresponding software packages have been developed rapidly, and a growing number of research analyses have been carried out on the microbiome, such as whole-genome shotgun (WGS), 16S, and the taxonomic profiling (Human Microbiome Project, 2012b), and have demonstrated significant associations between microbes and complex human diseases such as rheumatoid arthritis, colorectal cancer, obesity, and type 2 diabetes (Wang and Jia, 2016). However, these studies involve time-consuming and expensive biological experiments. Therefore, it is necessary to utilize the known information to predict the unknown microbe-disease interactions. Identifying microbe-disease interactions could promote discovering biomarkers and drugs for the prevention, diagnosis, treatment, and prognosis of diseases. Now, more and more computer algorithms (Chen and Zhang, 2013; Yang et al., 2014; Zhang et al., 2017; Zeng et al., 2018; Zhang et al., 2018a; Zhang et al., 2018b; Zhang et al., 2018c; Zhang et al., 2018d; Zeng et al., 2019) have been proposed for interaction prediction of miRNA-disease, lncRNA-disease, and drug-drug, and it is feasible to apply these methods to the microbe-disease association prediction field.

Recently, Ma et al. (2017) collected microbe-disease association data from previous published studies and constructed the Human Microbe-Disease Association Database (HMDAD). Based on the data from HMDAD, some microbe-disease association prediction methods have been proposed. Chen et al. (2017) used a KATZ measure to predict human microbe-disease association, and proposed an algorithm named KATZHMDA. KATZHMDA can predict new microbe-disease associations at a large scale. Bao et al. (2017) used network consistency projection and introduced an algorithm NCPHMD to predict human microbe-disease association. NCPHMD deals with unknown diseases or microbes that are not present in the disease-microbe databases. He et al. (He et al., 2018) presented an algorithm GRNMFHMDA. GRNMFHMDA assigns likelihood scores to unknown microbe-disease pairs by calculating weighted K nearest neighbor profiles of microbes and diseases, and then adapts the standard non-negative matrix factorization by integrating graph Laplacian and Tikhonov (L2) regularization to obtain a microbe-disease association prediction score matrix. Zou et al. (2017) designed an approach BiRWHMDA. BiRWHMDA constructs a heterogeneous network by connecting the microbe similarity network and the disease similarity network based on known microbe-disease associations, and then uses a bi-random walk to predict microbe-disease association.

In the paper, we propose a novel approach to predict potential micro-disease association based on the KATZ measure and bipartite network recommendation algorithm (KATZBNRA), which is an improvement on KATZHMDA (Chen et al., 2017). Similar to KATZHMDA, KATZBNRA uses the KATZ measure and the similarity of diseases and microbes according to the Gaussian interaction profile kernel to predict novel microbe-disease associations based on the known microbe-disease associations. Furthermore, in order to improve the predicting accuracy, KATZBNRA uses a bipartite network recommendation algorithm.

## Materials and Methods

### Known Disease-Microbe Associations

HMDAD (Human Microbe-Disease Association Database, http://www.cuilab.cn/hmdad) collected the curated human microbe-disease association data from microbiota studies where the microbes were determined by 16s RNA sequencing on the genus level (Ma et al., 2017). HMDAD provides public access to the data, and our known microbe-disease association data were downloaded from HMDAD. The data contains 450 distinct confirmed associations between 39 diseases and 292 microbes and is coded in an adjacency matrix , $A\;\epsilon \;R^{n_{d}\times n_{m}}$ where *n* *_d_* (or *n* *_m_*) is the number of diseases (or microbes). If there has been an experiment confirming that microbe *m* *_j_* relates to disease *d* *_i_*,*A*(*i,j*) is set to 1, otherwise *A*(*i,j*) is set to 0.

### Disease Gaussian Interaction Profile Kernel Similarity

According to (Chen et al., 2017), there is a generally accepted assumption that similar diseases show an interaction tendency to similar microbes. Similar to (Chen and Yan, 2013) and (Chen et al., 2017), we compute the disease network topologic similarity based on the Gaussian interaction profile kernel. For a disease-microbe association adjacent matrix *A*, the binary element *A*(*i,j*) at row *i* and column *j* encodes whether there is a confirmed association between disease *d*(*i*) and microbe *m*(*j*). The *i*th row of *A* is denoted by *IP*(*d*(*i*)). *IP*(*d*(*i*)) can be regarded a binary vector and is called the interaction profile of *d*(*i*) since it provides the association information of disease *d*(*i*) with all microbes. For two diseases, their similarity *KD*(*d* *_i_*,*d* *_j_*), based on the Gaussian interaction profile kernel, is calculated from their interaction profiles according to the following equations.

(1) $$KD(d_{i},d_{j})=\exp(-\gamma_{d}||IP(d_{i})-IP(d_{j})|{|}^{2})\;$$

(2) $$\gamma_{d}=\frac{\gamma_{d}^{'}}{\left(\frac{1}{n_{d}}\sum_{k=1}^{n_{d}}||IP(d_{k})|{|}^{2}\right)}$$

*KD*(*d* *_i_*,*d* *_j_*) is adjusted by the norm kernel bandwidth γ*_d_*, which is controlled by the bandwidth parameter ${\gamma^{\prime }}_{d}$. It is obvious that *KD*(*d* *_i_*,*d* *_i_*) = 1 and 0 < *KD*(*d* *_i_*,*d* *_j_*)≤1. According to (Vanunu et al., 2010), *KD* values in (0, 0.3] may be not informative, while *KD* values in [0.6, 1] may show significant similarity. Therefore, a logistic function transformation from *KD*(*x, y*) to *KD*'(*x, y*) in Equation (3) is utilized in order to measure the similarity of diseases *x* and *y* more appropriately.

(3) $$KD^{\prime }(d_{i},d_{j})=\frac{1}{1+e^{c*KD(d_{i},d_{j})+d}}$$

The parameters ${\gamma^{\prime }}_{d}$ and *c* could be set with cross-validation, but to simplify the calculation, we set ${\gamma^{\prime }}_{d}$ = 1 as in van Laarhoven et al., 2011, *c* = -15 as in (Vanunu et al., 2010). According to (Vanunu et al., 2010), we set *d* = *log*(9999) such that *KD′*(*d* *_i_*,*d* *_j_*) = 0.0001 when *KD*(*d* *_i_*,*d* *_j_*) = 0.

### Microbe Gaussian Interaction Profile Kernel Similarity

As mentioned before, similar diseases show an association tendency with similar microbes. To measure the similarity of microbes, we also used the Gaussian interaction profile kernel as before. It could be calculated in a similar way as follows.

(4) $$KM(m_{i},m_{j})=\exp(-\gamma_{m}||IP(m_{i})-IP(m_{j})|{|}^{2})$$

(5) $$\gamma_{m}=\frac{{\gamma^{\prime }}_{m}}{\left(\frac{1}{n_{m}}\sum_{k=1}^{n_{m}}||IP(m_{k})|{|}^{2}\right)}$$

where ${\gamma^{\prime }}_{m}$is also set to 1, and *IP*(*m* *_i_*) is the *i*th column of matrix *A*. Similarly, *KM'*(*m* *_i_* *, m* *_j_*) could be calculated as Equation (3). It should be noted that in each cross-validation experiment, the similarities of diseases and microbes will be recalculated (Sun et al., 2018).

### Bipartite Network Recommendation

The bipartite network recommendation is a two-step resource allocation process (Chen et al., 2018b), which is based on a bipartite graph *G*(*D, M, E*), where *D* represents disease nodes, *M* microbe nodes, *E* the edges corresponding to the known microbe-disease associations. Let *f*_0,_ *_i_*(*m* *_j_*) denote the initial resource allocated to a microbe node *m* *_j_* when considering disease *d* *_i_*, *k*(*m* *_j_*) be the number of adjacent disease nodes of microbe *m* *_j_*, and let *k*(*d* *_i_*) be the number of adjacent microbe nodes of disease *d* *_i_* in graph *G*.

When focusing on disease *d* *_i_*, each disease *d* *_i_* related microbe node is initially allocated with a resource value of 1, i.e. if there is an edge between the disease node *d* *_i_* and a microbe node *m* *_j_* in *G*, allocate an initial resource of 1 to *m* *_j_*. The first step of the bipartite network recommendation is to transfer the resource from microbe nodes to disease nodes according to Equation (6), and the second step is to transfer the resource of the disease nodes back to microbe nodes according to Equation (8).

(6) $$f_{1,i}(d_{l})=\overset{n_{m}}{\underset{i=1}{\sum }}\frac{a_{lj}f_{0,i}(m_{j})}{k(m_{j})}$$

where *a* *_lj_* is an element of matrix *A*, i.e. *a* *_lj_* = *A*(*l*, *j*) and

(7) $$a_{lj}=\{\begin{array}{c} 1,if\;disease\;d_{i}\;is\;related\;to\;microbe\;m_{j}\\ 0,otherwise\;\;\;\;\;\;\;\;\;\;\;\;\;\;\;\;\;\;\;\;\;\;\;\;\;\;\;\;\;\;\;\;\;\;\;\;\;\;\;\;\;\;\;\;\;\;\;\;\;\;\;\;\;\;\; \end{array}$$

In fact, *f*_0,_ *_i_*(*m* *_j_*) is also equal to *A*(*i*, *j*).

(8) $$f_{2,i}(m_{j})=\overset{n_{d}}{\underset{l=1}{\sum }}\frac{a_{lj}f_{1,i}(d_{l})}{k(d_{l})}$$

Equations (6) and (8) are integrated into Equation (9).

(9) $$f_{2,i}(m_{l})=\overset{n_{m}}{\underset{j=1}{\sum }}w_{lj}f_{0,\;i}(m_{j})$$

(10) $$w_{lj}=\frac{1}{k(m_{j})}\overset{n_{d}}{\underset{q=1}{\sum }}\frac{a_{qi}a_{qj}}{k(d_{q})}$$

**Figure 1:**
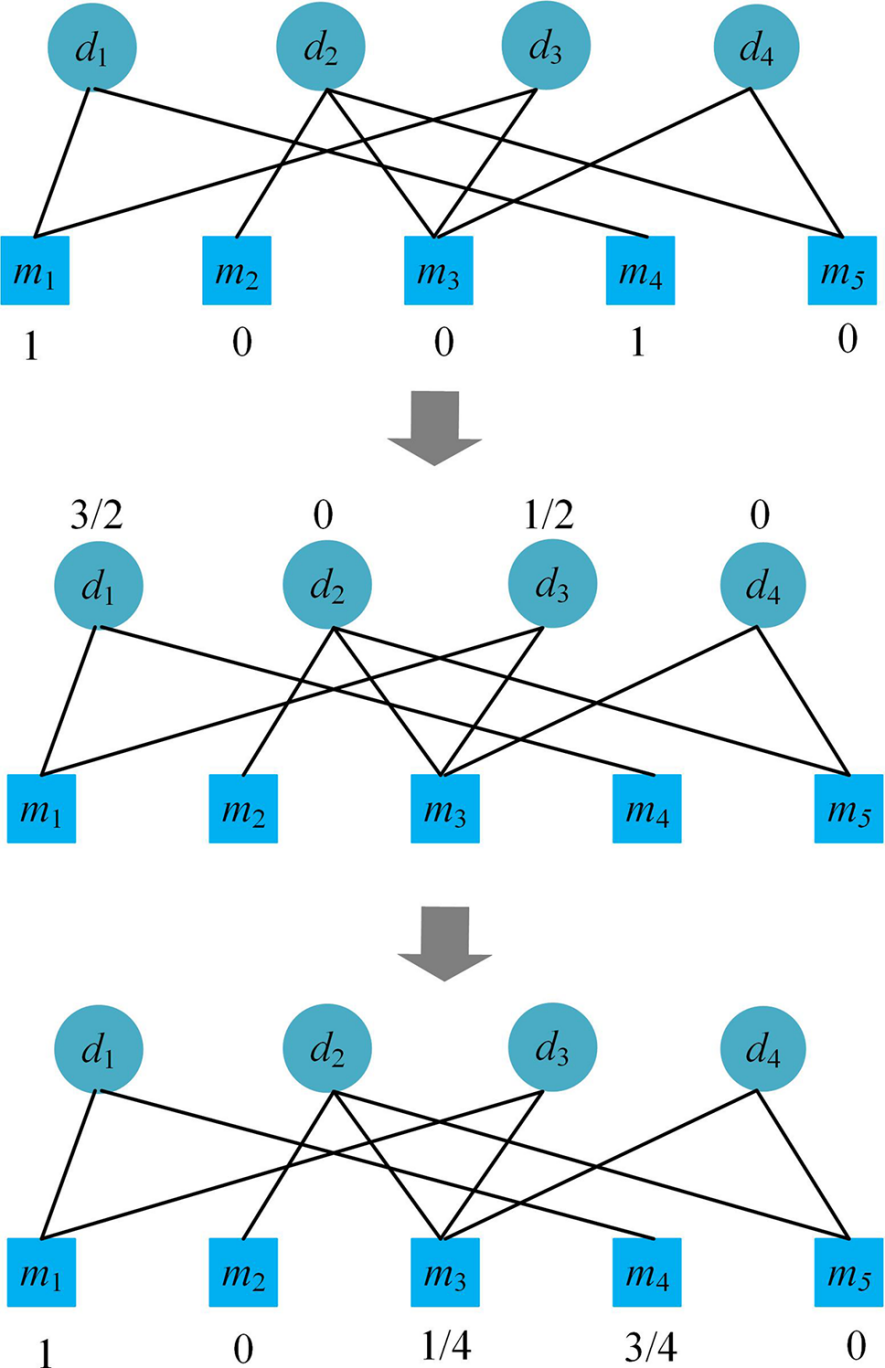
Illustration of the two-step resource-allocation process in a bipartite graph.

Please see an example of the process of the bipartite network recommendation focusing on disease *d*_1_ in **Figure 1**. After the process, we obtain the recommendation scores (1, 0, 1/4, 3/4, 0) of the microbes for disease *d*_1_, which suggests that besides *m*_1_ and *m*_4_, *m*_3_ may also be related to the disease.

The matrix form of Equation (9) is as follows.

(11) $$B=W\times A^{T}$$

where $W={\left\{w_{ij}\right\}}_{n_{m\times }n_{m}}$, and *B* is a matrix with *n* *_m_* rows and *n* *_d_* columns. The *i*th column of *B* is the recommend scores of bipartite network recommendation regarding disease *d* *_i_*

### KATZBNRA

**Figure 2:**
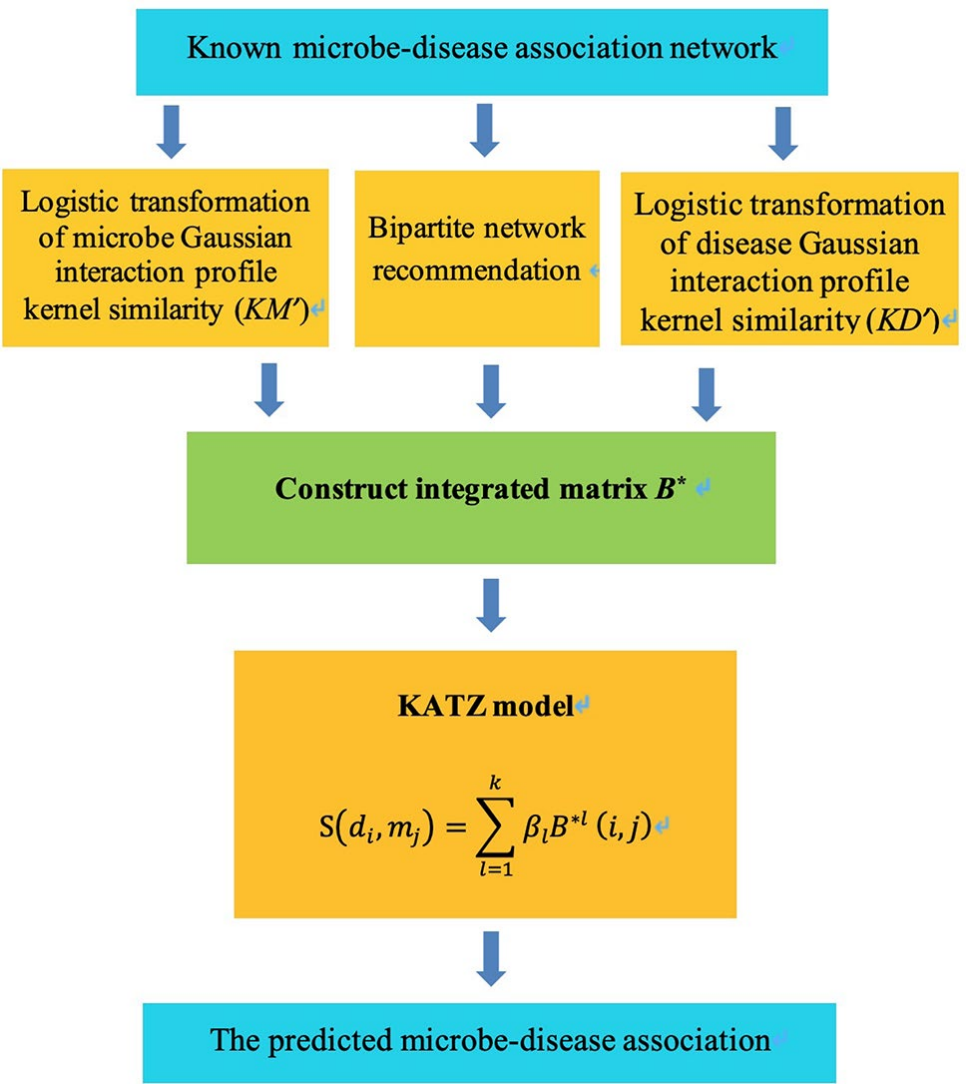
The diagram of KATZBNRA.

KATZBNRA uses the KATZ model to compute the associations between diseases and microbes and is illustrated in **Figure 2**. As a network-based computation method, the KATZ model (Chen, 2015) had been used in the problem of link prediction in the heterogenous network to calculate the similarity of nodes. There are two factors that have been regarded as effective similarity metrics in the KATZ model, the walk steps (length, i.e. the number of edges of the walk) and the number of walks from one node to another. We use the KATZ model to calculate similarities between the nodes of the microbe and disease by counting the number of walks between them. Here A*^l^*(*i,j*), the element of the *l*-th power of *A*, is the number of *l*-length walks between disease node *d* *_i_* and microbe node *m* *_j_*. Due to the limited data from HMDAD, matrix *A* is sparse. In order to use more information, we integrated the matrices *KM*, *KD*, *B* into a matrix B* as Equation (12) and replace *A* by B* in the KATZ model to calculate similarities between microbes and diseases.

(12) $$B^{*}=\left[\begin{array}{cc} KD^{\prime } & B\\ B^{T} & KM^{\prime } \end{array}\right]$$

Since walks between nodes of microbe and disease with different lengths have different contributions to similarities of node pairs, in order to dampen longer walks’ contribution, we introduced a parameter *β* *_l_* which is no smaller than 0, and if *l*_1_> *l*_2_, then $\beta_{l_{1}}<\beta_{l_{2}}$. The potential association between diseases *d* *_i_* and microbe *m* *_j_* can be calculated as follows.

(13) $$\text{S(}d_{i},m_{j}\text{)}=\overset{k}{\underset{l=1}{\sum }}\beta_{l}B^{*l}(i,j)$$

If k→∞, replace *β* *_l_* with *β* *^l^* (0<*β* <1) (Qu et al., 2018) and the matrix form of Equation (13) is as follows.

(14) $$\text{S}=\underset{l\geq 1}{\sum }\beta^{l}B^{*l}={\left(I-\beta B^{*}\right)}^{-1}-I$$

*S* is a matrix of size (*n* *_d_* + *n* *_m_*)×(*n* *_d_* + *n* *_m_*), and could be partitioned into four sub-matrices as shown in Equation (12).

(15) $$\text{S}=\left[\begin{array}{cc} S_{1,1} & S_{1,2}\\ S_{2,1} & S_{2,2} \end{array}\right]$$

where the rows of *S*_1,1_ and *S*_1,2_ are *n* *_d_*, the rows of *S*_2,1_ and *S*_2,2_ are *n* *_m_*, the columns of *S*_1,1_ and *S*_2,1_ are *n* *_d_*, and the columns of *S*_1,2_ and *S*_2,2_ are *n* *_m_*. The element *S*_1,2_(*i, j*) of *S*_1,2_ provides the possibility that an association between disease *d* *_i_* and the microbe *m* *_j_* exists, and our prediction result can be obtained from *S*_1,2_.

Considering that the walks of long lengths may be meaningless, we limit *k* in Equation (13) to be 2, 3 and 4, and the expression can be as follows.

(16) $$S_{k=2}=\beta \cdot B+\beta^{2}\cdot (KM^{\prime }\cdot B+B\cdot KD^{\prime })$$

(17)  Sk=3=Sk=2+β3⋅(B⋅BT⋅B+KM′2⋅B+KM′⋅B⋅KD′'+B⋅KD′2)

(18) $$\begin{array}{l} S_{k=4}=S_{k=3}\\ +\beta^{4}\cdot (K{M^{\prime }}^{3}\cdot B+B\cdot B^{T}\cdot KM^{\prime }\cdot B+KM^{\prime }\cdot B\cdot B^{T}\cdot B+B\cdot KD^{\prime }\cdot B^{T}\cdot B)\\ +\;\beta^{4}\cdot (B\cdot B^{T}\cdot B\cdot KD'+K{M^{\prime }}^{2}\cdot B\cdot KD'+KM'\cdot B\cdot KD^{'2}+B\cdot KD{'}^{3}) \end{array}$$

## Results

### Performance Evaluation

The test dataset of microbe-disease association was downloaded from HMDAD. We used LOOCV (leave-one-out cross validation), two-fold cross validation and five-fold cross validation to test the prediction performance of KATZBNRA on the HMDAD data.

In LOOCV, each known microbe-disease association takes turns to be picked out as the testing case and the other known associations are regarded as training data. We then obtained the prediction score of the test case output by KATZBNRA and ranked of the test case in the sorted list of all predicted microbe-disease associations in descending order of their scores. We used different thresholds to determine the correct predictions and wrong predictions and calculated corresponding FPR (false positive rate) and TPR (true positive rate) according to Equation (19). Finally, the results were presented in the ROC (receiver operating characteristics) curve plot of TPR against FPR.

(19) $$TPR=\frac{TP}{FN+TP}\;,\;\;FPR=\frac{FP}{TN+FP}$$

where FN is the number of false negative predictions (i.e. the cases whose prediction scores below the threshold), and TP is the number of true positive predictions (i.e. the cases whose prediction scores are not smaller than the threshold). FP is the number of the predicted associations that are not in the HMDAD dataset with scores not smaller than the threshold, and TN is the number of predicted associations that are not in the HMDAD dataset with scores smaller than the threshold. The area under a ROC curve is called AUC, and AUC is generally utilized to compare the power of predictive models. AUC of 0.5 indicates an entirely random prediction while AUC = 1 means a completely correct prediction.

In order to further test the prediction power of KATZBNRA, we also adopted 5-fold cross validation and 2-fold cross validation besides LOOCV. 5-fold (or 2-fold) cross validation randomly divides the microbe-disease associations equally into five (or two) parts and one of the five (or two) parts is reserved as the verification data while the remaining is used as training data. Considering the potential random sampling bias, we repeated each LOOCV, 2-fold and 5-fold cross validation test 100 times, and all ROC curves and AUCs are the average results of the 100 repeated tests. Meanwhile, we compared KATZBNRA with several state-of-the-art predictive methods using these validations.

**Figure 3:**
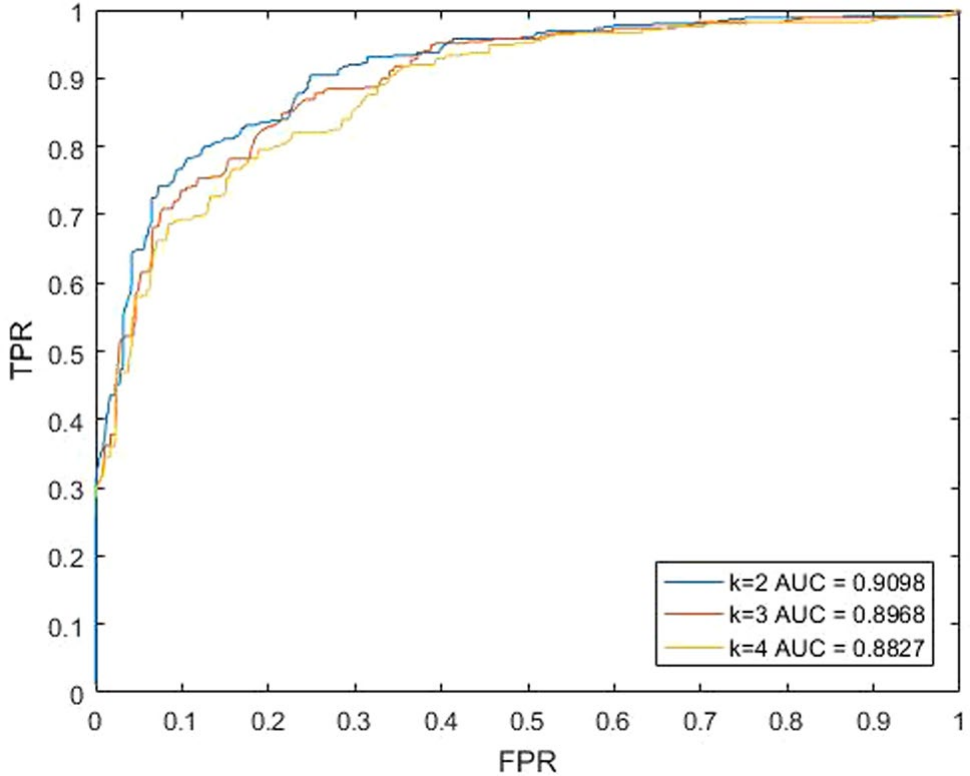
The predictive performances of KATZBNRA with different ks.

For our method KATZBNRA, the walk length *k* plays a critical role. To test the effect of *k*, we changed the value of *k*, and carried out a series of LOOCV experiments. As shown in **Figure 3**, when *k* is set to 2, 3 and 4, the AUCs of each walk lengths are 0.9098, 0.8968, and 0.8827, respectively. Obviously, when parameter *k* = 2, KATZBNRA achieved the best prediction performance and walks of longer lengths may make the association prediction worse. Therefore, in the following experiments, we set *k* = 2. KATZBNRA has two more parameters, γ′ and *β*. The test in a previous work (Chen et al., 2016) showed AUC tended to decrease when γ′ was increased from 1.0 to 1.5, 2.0 and 2.5, and β was increased from 0.01 to 0.05 and 0.1. We also evaluated the AUC of KATZBNRA with different values of parameter γ′ and c in Equation (2) and Equation (3), and the test results are shown in **Tables 1** and **2**, showing similar results as Chen et al., 2016. Therefore, we set γ′=1.0 and β=0.01.

**Table 1:** The AUC of KATZBNRA with γ′set different values.

*γ*′	AUC
1	0.9098
1.5	0.9083
2	0.9033

**Table 2:** The AUC of KATZBNRA with c set different values.

c	AUC
-15	0.9098
-10	0.9038
-5	0.8935

**Figure 4:**
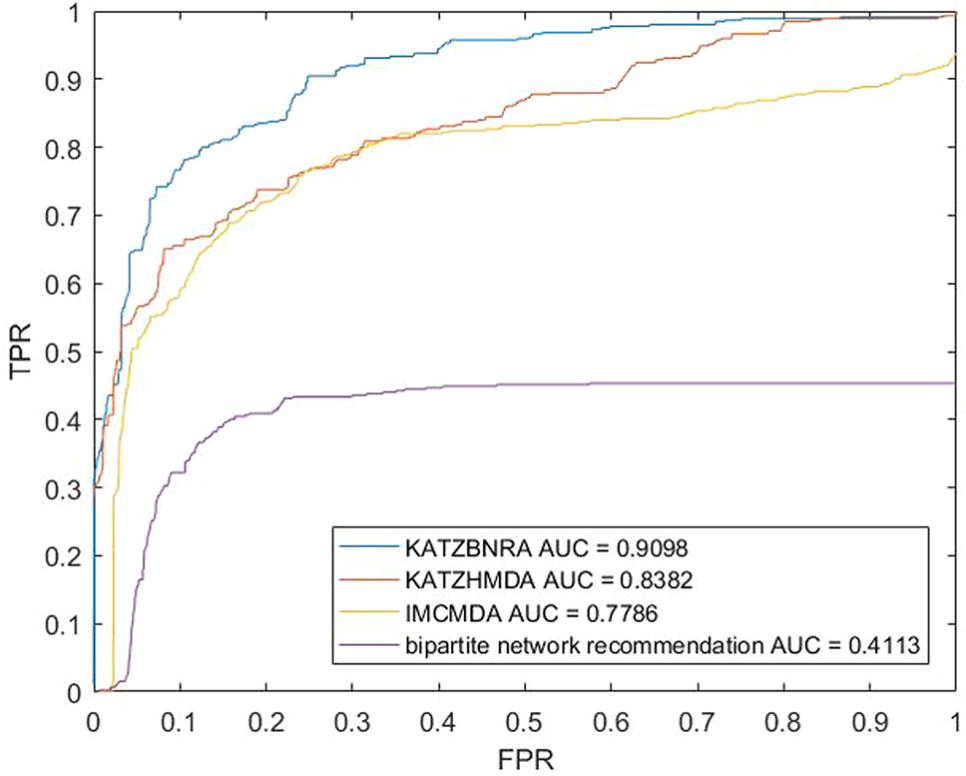
The LOOCV experimental results of KAZTBNRA, KATZHMDA, IMCMDA, and the native bipartite network recommendation.

**Figure 5:**
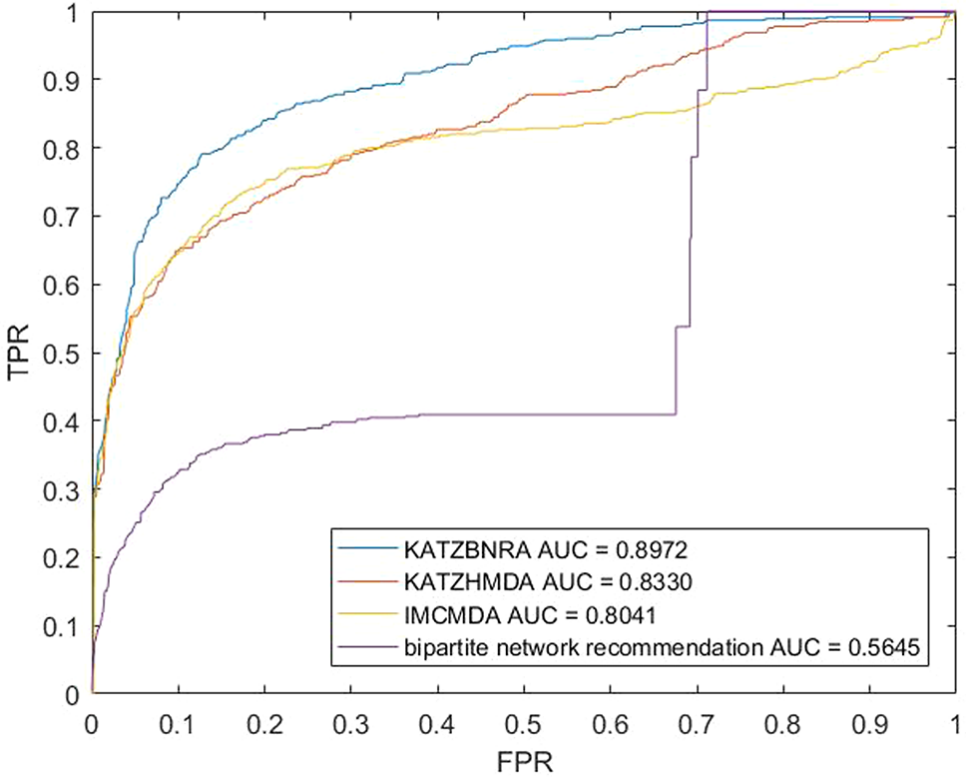
The 5-fold cross validation experimental results of KAZTBNRA, KATZHMDA, IMCMDA, and the native bipartite network recommendation.

**Figure 6:**
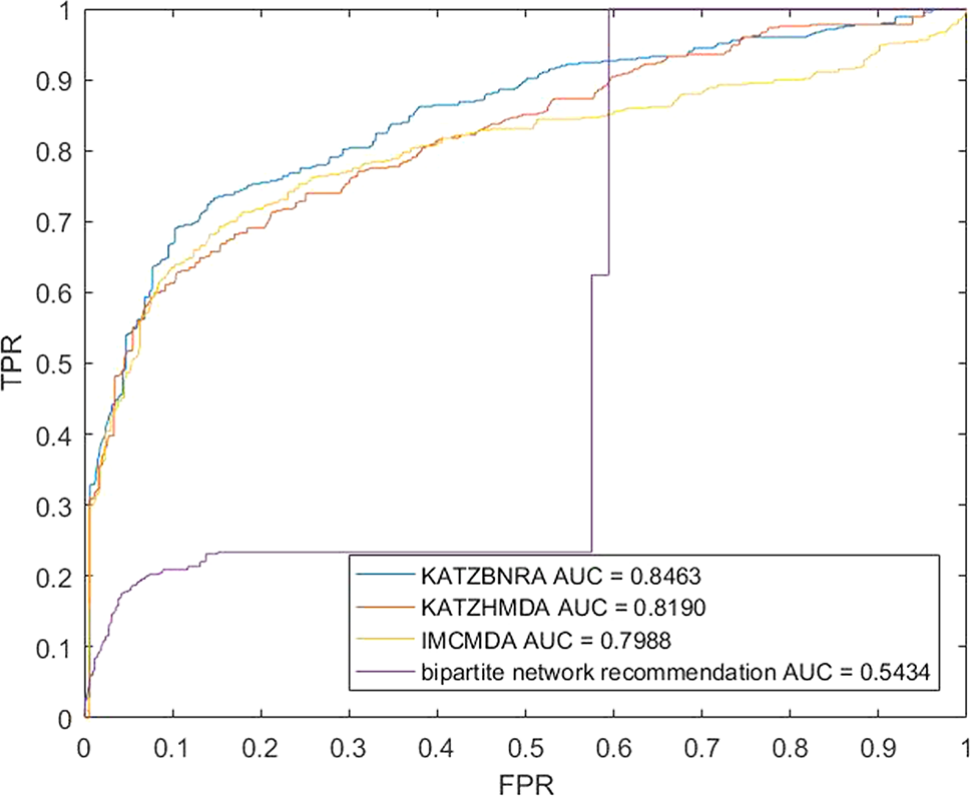
The 2-fold cross validation experimental results of KAZTBNRA, KATZHMDA, IMCMDA, and the native bipartite network recommendation.

We compared KATZBNRA with another three prediction methods, the native bipartite network recommendation (BNR) (Zhou et al., 2007), KATZHMDA (Chen et al., 2017), and IMCMDA (Chen et al., 2018a) using LOOCV, 5-fold cross validation and 2-fold cross validation. The global LOOCV showed that the AUCs of KATZBNRA, KATZHMDA, IMCMDA and BNR were 0.9098, 0.8382, 0.7786, and 0.4113, respectively, as shown in **Figure 4**−**6** show the 5-fold cross validation experimental results and the 2-fold cross validation experimental results, respectively. In 5-fold cross validation KATZBNRA, KATZHMDA, IMCMDA, and BNR obtained AUCs of 0.8972, 0.8330, 0.8041, and 0.5645, respectively, and in 2-fold cross validation, their AUCs were 0.8463, 0.8190, 0.7988 and 0.5434, respectively. In all the above experiments, the curves of KATZBNRA are above those of the other methods, which means that among the four methods, KATZBNRA achieved the best prediction performance.

### Case Studies

We studied asthma and inflammatory bowel disease (IBD) of microbe-related diseases of human beings based on recent published clinical and biological reports to further evaluate the ability of our method. The predicted disease-microbe associations which are contained in the HMDAD dataset are sorted according to their prediction scores in descending order. For asthma and IBD, we observed the microbes in the top 10 associations of the lists. This guarantees absolute independence between the verification candidate and the known association for model training.

As a common chronic lung inflammatory disease, asthma causes difficulty in breathing (Martinez, 2007). It is believed that asthma is caused by the environment and a combination of genes. For severe asthma, one of the leading causes is a microbe (Huang et al., 2011). All of top predicted 10 candidate microbes of KATZBNRA (**Table 3**) have been verified by recent studies.

**Table 3:** The Asthma-related microbe prediction of KATZBNRA. All of top 10 microbes were confirmed by recent studies.

Rank	Microbe	Evidence
**1**	Firmicutes	PMID: 23265859(Marri et al., 2013)
**2**	Actinobacteria	PMID: 23265859(Marri et al., 2013)
**3**	Clostridium coccoides	PMID:21477358(Vael et al., 2011)
**4**	Streptococcus	PMID: 17950502(Preston et al., 2007)
**5**	Lactobacillus	PMID: 20592920(Yu et al., 2010)
**6**	Lachnospiraceae	PMID:17433177(Rados et al., 2007)
**7**	Pseudomonas	PMID:13268970(Fein, 1955)
**8**	Burkholderia	PMID:24451910(Beigelman et al., 2014)
**9**	Fusobacterium	Dang et al., 2013(Dang et al., 2013)
**10**	Propionibacterium	PMID:27433177(Jung et al., 2016)

As a typical chronic GI (gastrointestinal) tract inflammatory bowel disease, IBD includes ulcerative colitis and Crohn’s disease (Lomax et al., 2006). We listed the top 10 IBD-related candidate microbes predicted by KATZBNRA in **Table 4**, among which eight microbes have been previously validated.

**Table 4:** Top 10 potential IBD-related microbes predicted by KATZBNRA

Rank	Microbe	Evidence
**1**	Clostridium coccoides	PMID:19235886(Sokol et al., 2009)
**2**	Firmicutes	PMID:25307765(Walters et al., 2014)
**3**	Bacteroidetes	PMID:25307765(Walters et al., 2014)
**4**	Staphylococcus	PMID:28174737(Pedamallu et al., 2016)
**5**	Prevotella	PMID:25307765(Walters et al., 2014)
**6**	Streptococcus	PMID:23679203(Kojima et al., 2014)
**7**	Propionibacterium	unconfirmed
**8**	Propionibacterium acnes	unconfirmed
**9**	Bacteroidaceae	PMID:17897884(Takaishi et al., 2008)
**10**	Haemophilus	PMID:24013298(Said et al., 2014)

## Discussion

Based on the bipartite network recommendation and the KATZ model, the paper introduced a novel disease-microbe association prediction method called KATZBNRA. KATZBNRA uses the Gaussian interaction profile kernel to calculate the similarity of diseases and microbes in the bipartite network containing the known microbe-disease associations from the HMDAD database, and the bipartite network recommendation score on the KATZ model enables KATZBNRA to predict potential disease-microbe associations with high accuracy. The experimental results of LOOCV, 5-fold cross validation, 2-fold cross validation and the IBD and asthma case studies have demonstrated the excellent and reliable prediction ability of KATZBNRA. With regard to similar prediction problems such as predicting lncRNA-disease, drug-target, gene-disease, miRNA-disease, and other biological associations, this model can be applied with small modifications.

## Data Availability Statement

Publicly available datasets were analyzed in this study. This data can be found here: http://www.cuilab.cn/hmdad.

## Author Contributions

SL and MX conceived the study and the conceptual design of the work. SL and XL collected the data and implemented the KATZBNRA algorithm. SL tested the algorithms and drafted the manuscript. MX and XL polished the manuscript. All authors have read and approved the manuscript.

## Funding

This work is supported by the National Natural Science Foundation of China (Grant No. 61772197).

## Conflict of Interest

The authors declare that the research was conducted in the absence of any commercial or financial relationships that could be construed as a potential conflict of interest.
